# Breast Cancer Metastasis Developed Inside an Angiomatous Meningioma: A Case Report

**DOI:** 10.7759/cureus.67578

**Published:** 2024-08-23

**Authors:** Amira Kamel, Ligia G Tataranu, Diana Pasov, Dan Paunescu, Radu Eugen Rizea

**Affiliations:** 1 Neurosurgery, Emergency Clinical Hospital Bagdasar-Arseni, Bucharest, ROU; 2 Neurosurgery, University of Medicine and Pharmacy "Carol Davila", Bucharest, ROU; 3 Pathology, Emergency Clinical Hospital Bagdasar-Arseni, Bucharest, ROU

**Keywords:** cancer, pathology, metastasis, breast cancer, meningioma

## Abstract

The intracranial development of breast metastasis inside a meningioma is a rare entity known as tumor-to-tumor metastasis or tumor-to-meningioma metastasis. Although rare such cases have been reported in the scientific literature, most of them are from breast and lung cancer, and even rarer from the genitourinary tract, while meningioma is the most cited to harbor these metastases. A thorough histopathological analysis represents the cornerstone of the diagnosis of these lesions. The current article presents a case of breast cancer metastasis developed inside a meningioma in a female patient, which is a rare metastatic presentation. Although hormonal causes have been incriminated, the pathophysiology of this phenomenon is still unclear.

## Introduction

The development of a metastatic tumor within another primary neoplasm is widely known in the scientific literature as tumor-to-tumor metastasis or tumor-to-meningioma metastasis (TMM) and represents a very rare entity [1]. The most common harboring tumors are represented by meningiomas, and the most frequent metastatic tumors are represented by breast and lung carcinomas [1]. However, rare cases of other recipient tumors have been reported, such as hemangioblastomas, astrocytomas, ependymoma, oligodendroglioma, acoustic neuroma, and even secondary lymphoma [2, 3], and uncommon metastases from prostate, gastric, genitourinary, thyroid, and parotid gland neoplasms were reported [3-5]. It has been stated that the prevalence of this pathology increases with age and that there is no sex preference [6].

The first case of TMM was reported in 1930 by Fried in an article that described a metastasis of bronchogenic carcinoma inside a meningioma [7]. Since then, less than 100 cases have been reported worldwide [6].

The current study aims to report a case of breast cancer TMM in a 71-year-old patient to contribute to a deeper understanding of this rare entity.

## Case presentation

Our female patient was diagnosed for the first time in 2009 with a left breast lesion with a malignant aspect. After the unilateral left mastectomy, the final diagnosis was of invasive ductal carcinoma of the breast, and adjuvant chemotherapy followed. The oncological follow-up was negative for four years, until 2013, when a similar right breast lesion was diagnosed. The following procedures were similar, with unilateral right mastectomy and a diagnosis of invasive ductal carcinoma of the breast, followed by adjuvant chemotherapy. Later, in 2017, the oncological follow-up came across a femoral bone breast cancer metastasis with a femoral neck fracture that was surgically treated (hip replacement implant as well). However, this was not the only metastasis, as another two pulmonary lesions were discovered, although the surgical removal was refused by the patient, and there was no histopathological diagnosis.

In 2022, five years after her last diagnosis, at 71 years old, she was referred to our department for neurological symptoms that progressively aggravated in the last three months, especially the headaches that were more intense in the last few days. Other than this, the medical history of the patient revealed ischemic cardiomyopathy, arterial hypertension, dyslipidemia, anemia, class 1 obesity, surgically treated bilateral cataracts, and nephroangiosclerosis.

**Figure 1:**
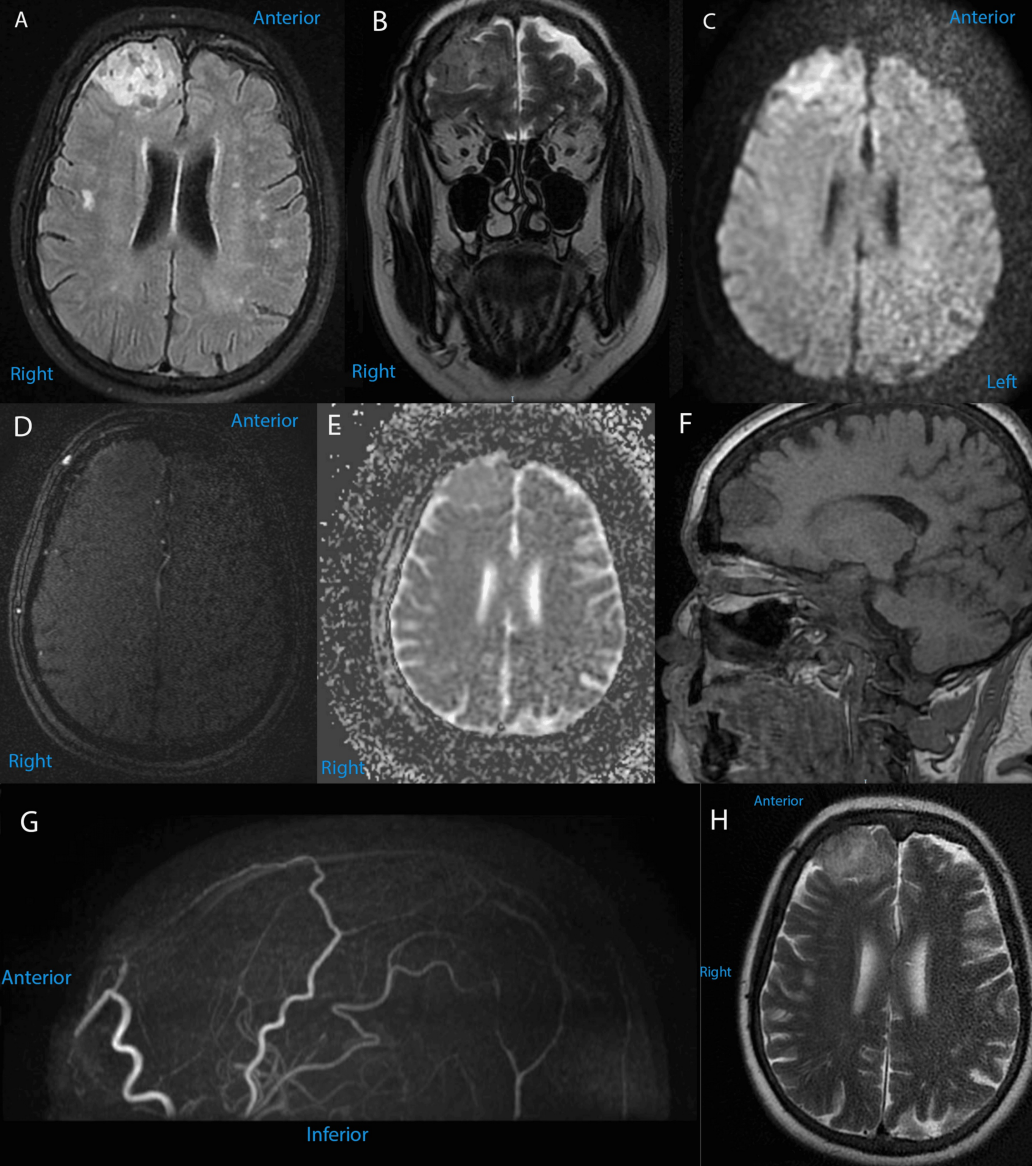
Preoperative MRI brain images of our patient, with different sequences. (A) Axial FLAIR sequence; (B) coronal T2 FSE; (C) axial diffusion-weighted scan (b1000); (D) axial 3D TOF; (E) axial ADC; (F) sagittal 3D T1; (G) sagittal (cross-section) 3D TOF MR angiogram; (H) axial T2 propeller. FLAIR, fluid-attenuated inversion recovery; FSE, fast spin echo; TOF, time-of-flight

At admission, the patient complained of severe refractory headaches, vertigo, hypomnesia, and hypoprosexia, and the Karnofsky Performance Status Scale at admission was 80. The brain MRI performed at admission in our institution revealed a right frontal well-circumscribed extra-axial tissue mass adjacent to meninges, with an anteroposterior diameter of 3 cm, craniocaudal diameter of 2 cm, and transverse diameter of 4 cm. The lesion was T1-hypointense, T2/FLAIR-hyperintense, with discreet inhomogeneity due to various focal areas that were T2/FLAIR-hypointense. The tissue mass also has a degree of diffusion restriction. No vascular structures were described inside or near the lesion on 3D TOF, and no adjacent edema was observed (Figure 1).

The preoperative diagnosis was of cerebral meningioma. Subsequently, a neurosurgical intervention was performed through a frontal craniotomy, with a Simpson grade II resection (complete tumor removal with coagulation of dural attachment). The intraoperatively gross findings revealed a well-circumscribed tumor of gray-white color, polylobate, solid in consistency, relatively homogeneous, well vascularized, connected both arterially and venously to the vascular network of the right frontal cortex, with dimensions of about 40/30/20 mm.

Postoperatively, our patient underwent radiotherapy to prevent recurrences. No postoperative deficits or complications were recorded, and no symptoms of headaches and vertigo were present at discharge. The hypomnesia and hypoprosexia symptoms significantly improved in the postoperative settings, and mental examination at two months follow-up revealed almost normal results.

Pathological findings

**Figure 2:**
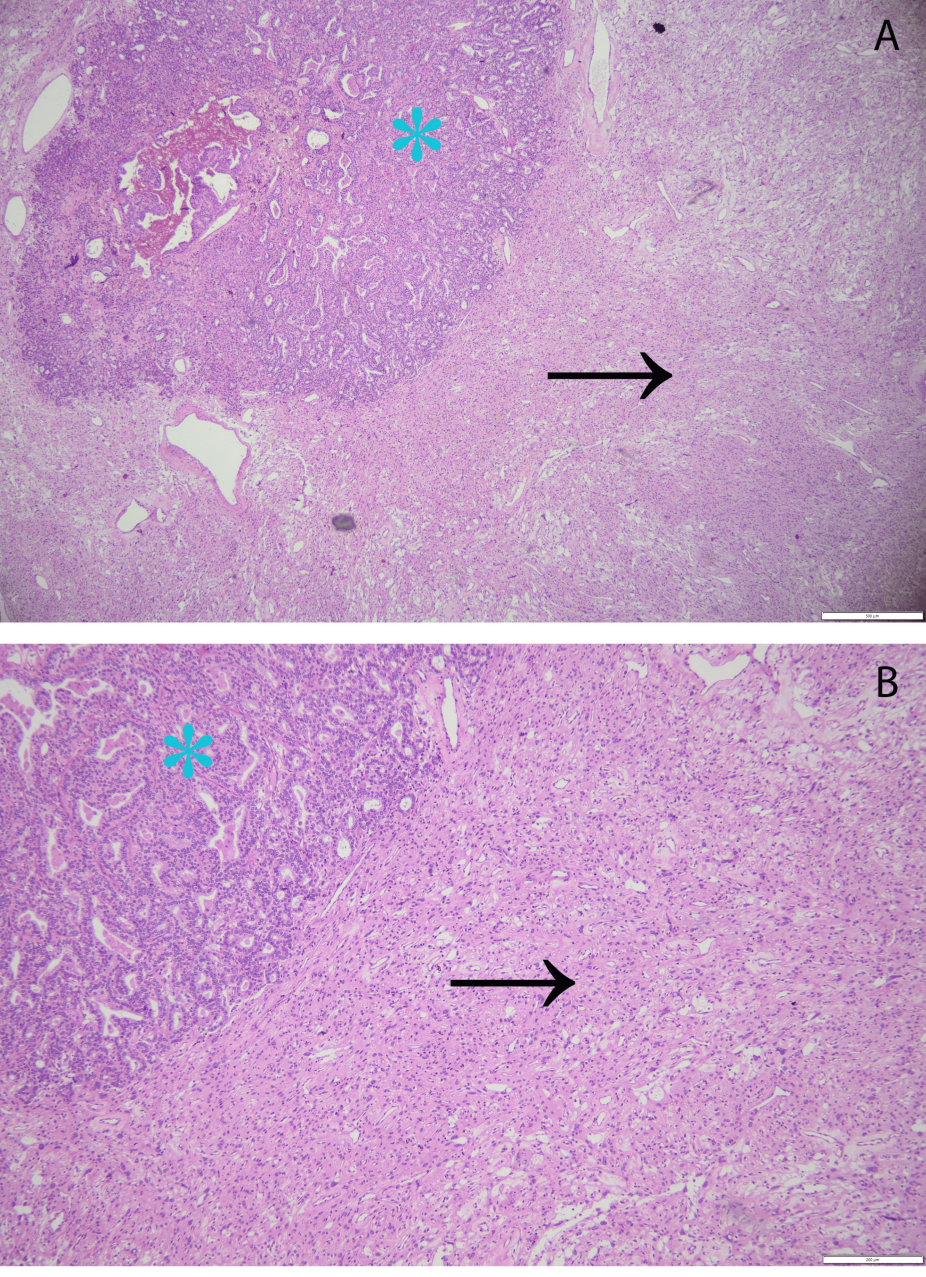
Photomicrograph representing the collision between an angiomatous meningioma WHO grade 1 and a tumoral nodule of tubular structure carcinoma - hematoxylin and eosin staining. (A) Magnification 4x, angiomatous meningioma (black arrow) and carcinoma (blue asterisk); (B) magnification 10x, angiomatous meningioma (black arrow) and carcinoma (blue asterisk).

**Figure 3:**
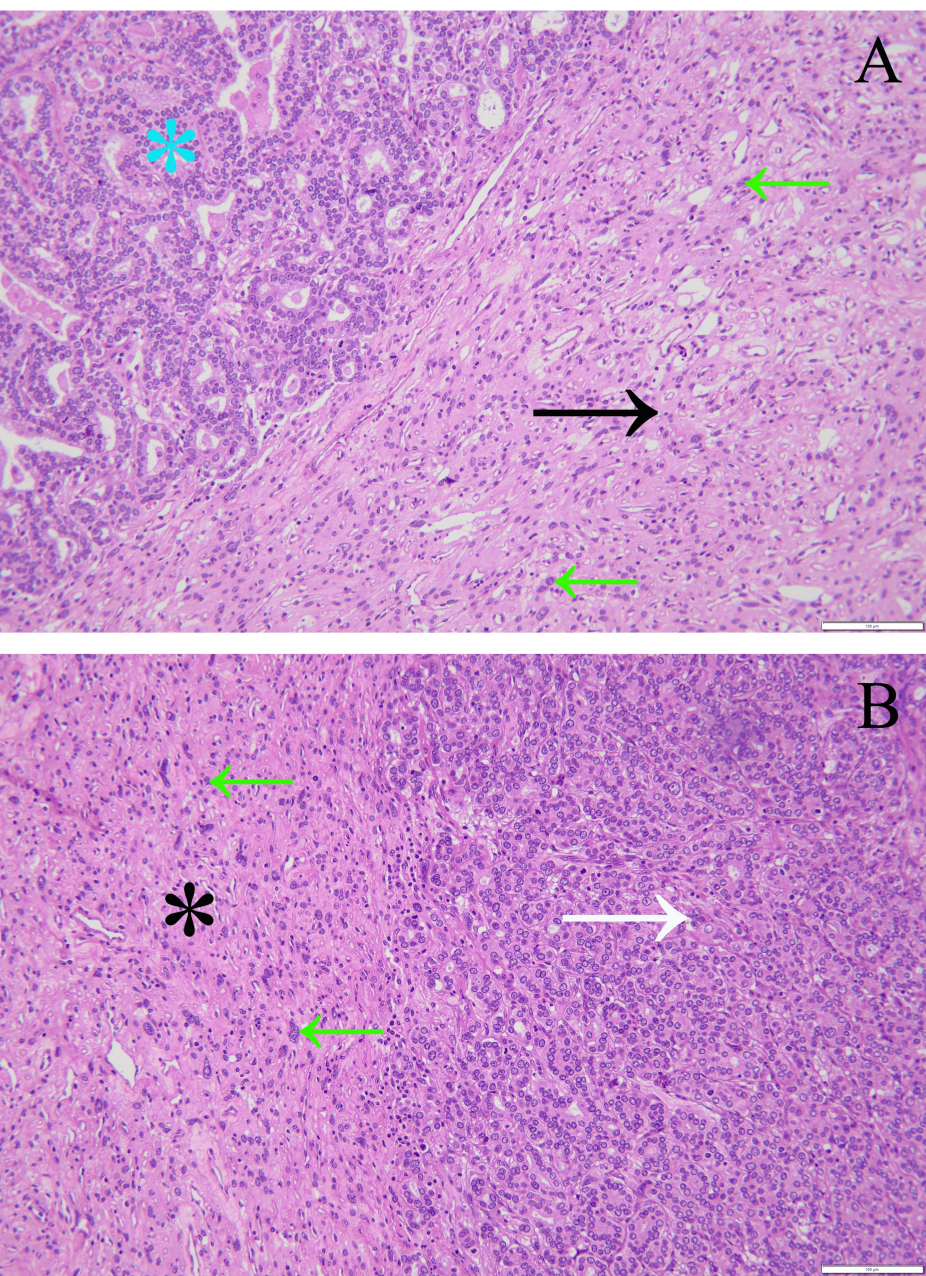
Magnification on a greater scale of the same tumoral sample, stained hematoxylin-eosin Both images have the same magnification, although they are different parts of the tumoral sample. (A) Magnification 20x. The black arrow shows abundant blood vessels and meningothelial cells that express numerous degenerative nuclear atypia, while the blue asterisk shows a very different carcinomatous tissue; the green arrows reveal meningothelial cells forming syncytium. (B) Magnification 20x. The black asterisk reveals the tumoral benign tissue, while the white arrow shows a predominant pattern composed of ovoid tubules of small dimensions, with open lumina, lined by an epithelial cells layer. Similar to (A), the green arrows show meningothelial cells. The image pointed by the white arrow is compatible with a carcinoma. Contributed by Pasov Diana.

The surgical material consisted of various tumoral pieces that revealed different tissue patterns, and the predominant pattern was represented by the meningioma. Noteworthy were the syncytial cells with indistinct cellular membranes and eosinophilic cytoplasm. The vascular component exceeds half of the total examined area. These features were consistent with a benign angiomatous meningioma WHO grade 1. Multiple regions of tubular structure carcinoma were observed intratumorally, which represent metastases from breast carcinoma, also given the clinical context of the patient. Angular glands with cytoplasmic snouts were also noted on the neoplastic tissue, especially on 4x and 10x magnifications (Figures 2-3).

The final diagnosis based on histological examination of the neurosurgical specimen was predominantly of angiomatous meningioma with a microvascular pattern and small areas of microcystic meningioma, with multiple intratumoral areas of tubular structure carcinoma.

The results from the immunohistochemistry analysis confirmed the diagnosis mentioned earlier. The microscopic description was of a tumor fragment represented by proliferation of meningothelial type with angiomatous and microcystic pattern presenting multiple foci of malignant microscopic tumor infiltration of epithelial type arranged tubular-glandular and papillary having the microscopic appearance of NOS ductal breast carcinoma with an intermediate grade of G2 anaplasia.

After antibody testing (rabbit monoclonal), the results revealed GATA3 positivity in tumor cells, ER-positive nuclear expression in over 90% of tumor cells, negative PgR nuclear expression, Human Epidermal Growth Factor Receptor 2 (HER2) negativity with a score of 1 according to the American Society of Clinical Oncology/College of American Pathologists (ASCO/CAP) 2018 revised criteria, a Ki-67-positive nuclear proliferation index in approximately 20% of tumor cells, and TTF1 negativity.

Finally, the immunohistochemistry staining concluded that the brain metastasis originated from ductal breast carcinoma NOS (WHO/IARC ICD-O: 8500/6), with a grade 2 histological malignancy (Nottingham score 6). The immunohistochemical profile was: ER-positive, PgR-negative, and HER2-negative, with a Ki-67 index of 20%, and the metastasis was located in the meningioma.

## Discussion

The coexistence of different tumors in the same patient is not uncommon; however, the coexistence of histopathologically distinct intracranial tumors is rare. The scientific literature described the development of a metastasis inside a meningioma as tumor-to-tumor metastasis or tumor-to-meningioma metastasis (TMM), and it has been stated that meningiomas are the most common types of tumors able to harbor metastases, most frequently arising from breast or lung carcinomas [8].

Although the exact pathophysiology of this rare phenomenon has not been completely elucidated, various theories have been proposed to explain it. One of the main theories incriminates the hypervascularity of meningiomas, which is usually a typical feature compared to other intracranial neoplasms. In addition to hypervascularity, meningiomas have low metabolic activity, with a slow development process and an indolent feature. All these characteristics create nutritive conditions for metastases to develop and grow [9,10].

Sex hormone imbalances were also cited in the pathophysiology of TMM development. An association between meningioma and breast cancer was described in a recent systematic review and meta-analysis by Degeneffe et al. [11]. The authors of the study concluded that female patients with meningiomas have 10-fold higher odds of developing breast cancer and female patients with breast cancer have 1.42-fold higher odds of developing meningiomas during their lifetime [11]. The link between these two pathologies and sex hormones was suggested in the last decades mainly by the higher incidence in women, the expression of a high amount of receptors for estrogen, progesterone, and androgens, as well as the therapeutic response after hormone therapy, and the effect of breastfeeding in these patients [12].

Besides the hypervascularity and sex hormone imbalances, the theory of intracellular communication has been studied. E-cadherin is a frequently described molecule involved in the developing processes of TMM, especially in ductal breast carcinoma. The main role of this cell-adhesion molecule is to facilitate signal transduction and tumoral growth [13]. Furthermore, the overexpression of an oncogene known as c-myc has been incriminated as an important factor involved in the development of meningioma and breast cancer, not only when these pathologies coexist but also when they arise separately [8].

TMM remains a rare entity, with less than 100 cases described in the worldwide scientific literature, and 4 criteria must be met to diagnose it [2]. The first one regards the primary tumor and says that at least two primary tumors must exist. In our case, this criterion has been met. Our patient was diagnosed with bilateral invasive ductal carcinoma of the breast. First, she was diagnosed with left breast cancer, and she underwent surgery, and after four years she was again diagnosed with breast cancer, this time on the right side, and she was surgically treated again. Four years after the last intervention, she was diagnosed with femoral bone metastasis that was successfully operated on. She was also diagnosed with two small pulmonary metastases, but this time she refused the treatment. However, she was also diagnosed with a second tumor which was a frontal angiomatous meningioma with a meningothelial component. This diagnosis was obtained after neurosurgical excision of the lesion in our department.

The second criterion to diagnose a TMM states that the harboring tumor must be a true neoplasm, which was also the case for our patient, whose recipient tumor was an angiomatous meningioma.

Another important aspect of the diagnosis of TMM is represented by the location of metastatic growth, and this is the third criterion. The development of the metastasis must be inside of the harboring tumor, not adjacent; otherwise, the phenomenon would be called collision, which is a different entity from TMM.

The last criterion for diagnosis excludes metastases in lymph nodes that were already present, so our patient met all the criteria for TMM.

The differential diagnosis of TMM is primarily made with collision tumors. Metastases to leptomeninges may develop near the meningioma with subsequent tumoral fusion that is known as collision [3]. To differentiate collision tumors from TMM, other criteria have been established by Pamphlett[14]. First, to diagnose a TMM, the metastatic focus must be at least partially enclosed by a rim of histologically distinct host tumor tissue, which is the case for our patient, and second, the primary carcinoma must be demonstrated and must be the same as the metastasis [14]. Given the fact that our patient has met all the criteria, we concluded that the final diagnosis was breast cancer TMM.

## Conclusions

Neurosurgeons must be aware of the possibility of TMM in patients with specific clinical contexts, to provide the best surgical planning, as particular areas for tumoral biopsy should be considered. While pieces of information can be obtained from neuroimaging studies, these are insufficient for a diagnosis, and the histological analysis of the tumoral tissue sample makes the final contribution. The correct diagnosis is crucial, given the fact that a meningioma has a different management from a carcinoma. Although the exact pathophysiology of this phenomenon remains unclear, several plausible theories have been proposed. We hope that this case report will contribute to a better understanding of TMM and aid in developing optimal management strategies for the disease
